# Une cause rare et méconnue de douleur abdominale: l’appendagite épiploïque: à propos d’un cas

**DOI:** 10.11604/pamj.2018.31.87.14604

**Published:** 2018-10-04

**Authors:** Siham Alaoui Rachidi

**Affiliations:** 1 Service de Radiologie, Centre Hospitalier Provincial, Taounate, Maroc

**Keywords:** Douleur abdominale, diagnostic, tomodensitométrie, Abdominal pain, diagnosis, CT scan

## Abstract

Les appendagites épiploïques primitives sont des causes rares d’abdomen aigu. Elles sont souvent prises pour une appendicite aiguë ou une sigmoïdite diverticulaire et le diagnostic est posé au cours d’une intervention chirurgicale. Nous rapportons un cas où la tomodensitométrie a permis de poser le diagnostic et nous insisterons sur l’aspect imagerie qui permet de sursoir ainsi à une chirurgie inutile.

## Abstract (en)

Primitive epiploic appendagites are a rare cause of acute abdomen. They often mimic acute appendicitis or diverticular sigmoiditis. Diagnosis is made during surgery. This study aims to higlight the key role of CT scan in the diagnosis of epiploic appendagitis enabling patients to avoid unnecessary surgery.

## Introduction

L’appendagite épiploïque est une inflammation primitive d’un appendice épiploïque. Cet événement pathologique considéré comme rare, a longtemps été exceptionnellement diagnostiqué en période préopératoire. Les progrès de l’imagerie médicale permettent, désormais, d’éviter des interventions chirurgicales inutiles. C’est ce qu’illustre l’observation présentée ci-dessous.

## Patient et observation

**Figure 1:**
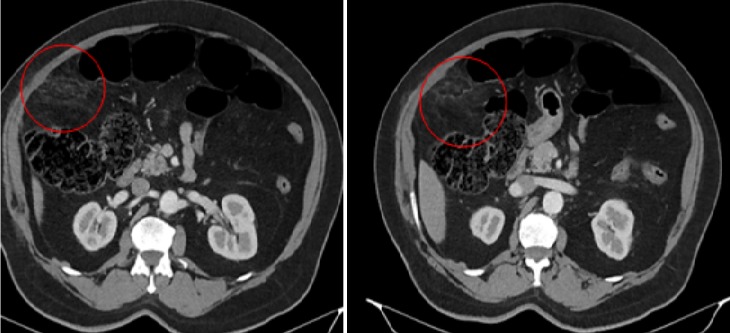
TDM abdominale: formation ovoïde intra-abdominale à centre graisseux, angio-centrée, bien limitée en sous hépatique avec densification périphérique correspondant à une appendagite

**Figure 2:**
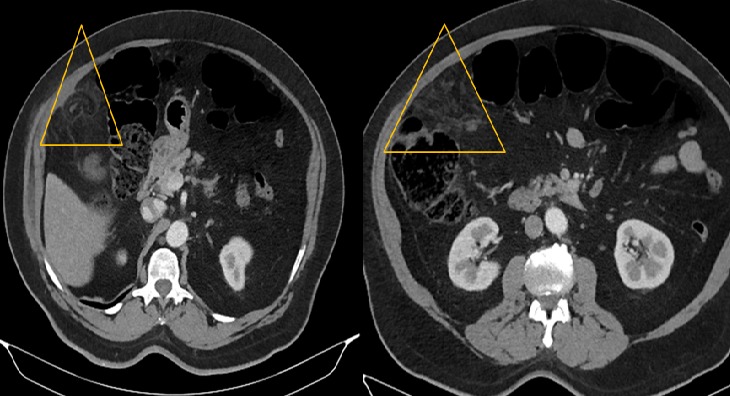
TDM abdominale en coupes axiales après injection du produit de contraste montrant formation graisseuse en navette sous-pariétale ayant une densité légèrement supérieure à la graisse normale, cernée d’un liseré hyperdense, associée à une infiltration de la graisse périphérique

**Figure 3:**
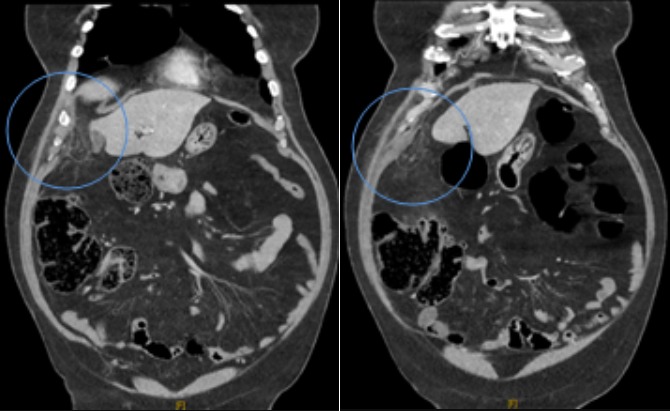
TDM abdominale en reconstruction coronale: mise en évidence d’une lésion d’aspect nodulaire avec une infiltration de la graisse sous hépatique para-colique

**Figure 4:**
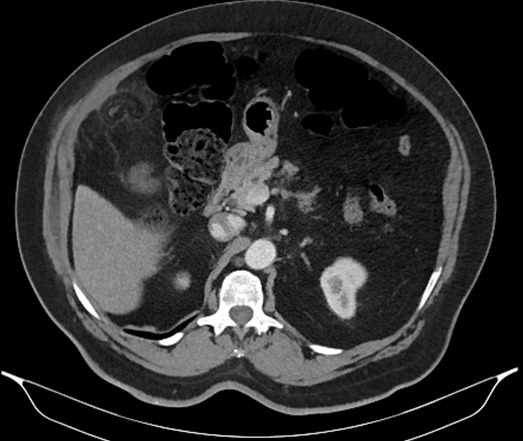
TDM abdominale objectivant une image en “navette” sous forme de lésion de densité graisseuse sous hépatique, bien limitée par un fin liseré de densité plus élevée réalisant un aspect en “ring sign”

**Figure 5:**
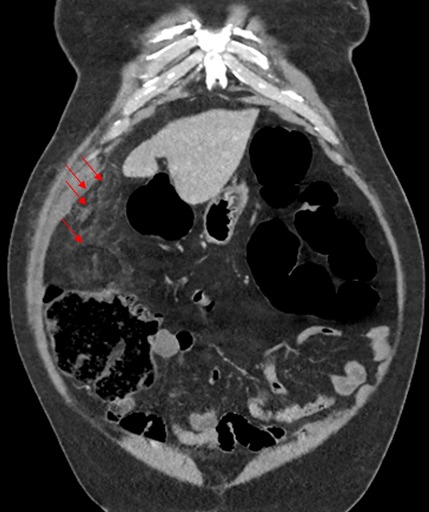
TDM abdominale montrant une appendagite avec un épaississement péritonéal au contact

B.M agé de 76 ans, sans antécédants pathologiques notables, admis pour des douleurs de l’hypochondre droit évoluant depuis six jours et d’aggravation progressive. Il ne souffrait ni de troubles du transit, ni de signes fonctionnels urinaires associés et était apyrétique. La palpation abdominale mettait en évidence une douleur exquise très localisée, superficielle, siégeant au niveau du flanc et de la fosse iliaque droite. Sur le plan biologique, il présentait un syndrome inflammatoire avec hyper leucocytose (12000/mm^3^), CRP augmentée (62 mg/l). L’examen cytobactériologique des urines était négatif. La radiographie de l’abdomen sans préparation ne mettait en évidence ni niveau hydroaérique, ni calcification en projection des voies urinaires. La tomodensitométrie révélait une infiltration et densification focale de la graisse epiploique sous hépatique formant une image ovalaire centre par des structures linéaires d’allure vasculaire. Cette anomalie est mitoyenne au colon qui est normale (Figure 1, Figure 2, Figure 3, Figure 4, Figure 5). Le diagnostic d’une appendagite épiploique a été retenu sur la base de données scannographiques. Le traitement était médical: il comprenait essentiellement des antalgiques et des anti-inflammatoires. Les symptômes ont régressé au bout d’une semaine en moyenne.

## Discussion

L’appendagite est une étiologie de douleur abdominale peu commune chez l’adulte et exceptionnelle chez l’enfant [1]. Il s’agit de l’inflammation des franges graisseuses épiploïques. Les appendices épiploïques sont des formations graisseuses recouvertes de péritoine et contenant des vaisseaux issus de la vascularisation colique. Leur vascularisation précaire issue des branches artérielles coliques et leur morphologie pédiculée les prédisposent à des phénomènes de torsion et d'ischémie. Ils sont pédiculés à partir de la séreuse colique du cæcum jusqu'à la jonction recto-sigmoïdienne et de topographie antérieure et latérale par rapport au côlon. Ils sont au nombre d'une centaine au total, d'une taille variant entre 0,5 et 5 cm. Ils sont plus nombreux sur le sigmoïde et le cæcum, où ils sont également plus volumineux. Ils se répartissent en deux lignes le long des bandelettes coliques et ils n'ont pas de fonction définie connue [2, 3]. Ils seraient plus nombreux et plus volumineux chez les patients obèses [1, 4, 5].

Sur le plan physiopathologique, deux mécanismes sont décrits [6, 7]: soit la torsion qui est facilitée par leur grande mobilité dans la cavité péritonéale et leur caractère pédiculé, les appendices graisseux peuvent subir des mécanismes de torsion favorisant la possibilité d’infarcissement; soit la thrombose spontanée de la veinule centrale.

Concernant la clinique, l’appendagite épiploïque peut survenir à tout âge, avec une légère prépondérance pour les hommes d’âge moyen, entre 40 et 50 ans [8]. L’obésité semble être le seul facteur de risque reconnu [7]. La douleur, d’apparition brutale, est permanente et focale, pouvant être montrée du doigt par le patient [6]. Par ordre de fréquence croissante, elle prédomine aux fosses iliaques droite et gauche où les appendices sont les plus volumineux. En général, il n’y a pas de défense abdominale et le transit reste présent. Les signes d’accompagnement à type de nausées, vomissements, constipation ou diarrhée sont rares. Le patient est quelques fois subfébrile. Très rarement, l’appendagite épiploïque peut s’incarcérer dans une hernie inguinale et se présenter sous la forme d’une masse inguinale douloureuse [3]. La biologie est évocatrice avec une NFS normale, sans hyperleucocytose, mais avec une élévation isolée de la CRP [2].

L’exploration TDM, permettant de faire le diagnostic de certitude, se traduit par une image caractéristique de masse avec une densité légèrement supérieure à la graisse normale (environ -80 U Hounsfield le plus souvent), limitée par un anneau apparaissant hyperdense après injection de produit de contraste et correspondant à l’inflammation de la séreuse et réalisant l’aspect dit du « ring sign » [5, 7, 8]. Moins fréquemment, on peut distinguer un centre hyperdense dont l’origine pourrait correspondre à une fibrose septale ou à une thrombose vasculaire. Il peut également y avoir un effet de masse sur les anses digestives adjacentes, un épaississement du péritoine au contact ainsi qu’un épaississement de la paroi colique en regard [8].

En imagerie par résonance magnétique, la morphologie de l’appendagite épiploïque est similaire. La zone concernée suit le signal de la graisse à l’exception de l’infiltration périphérique et de l’anneau qui exprime la composante inflammatoire de la séreuse et qui se rehausse après injection de Gadolinium [9]. L’aspect échographique le plus souvent observé chez l’adulte est celui d’un nodule, non compressible et antérieur par rapport au colon [10]. L’étude en doppler couleur révèle une absence ou une baisse de flux au sein de la masse et une hypervascularisation périphérique. Le traitement préconisé est strictement conservateur [5, 7, 8]. Ainsi, les appendagites épiploïques primitives traitées symptomatologiquement par antalgiques et anti-inflammatoires non stéroïdiens régressent cliniquement en moins d’une semaine, 5 jours en moyenne [4].

## Conclusion

Malgré son caractère rare, l’appendagite est un diagnostic à connaître. Son aspect radiologique est caractéristique et son diagnostic permet d’éviter une chirurgie abdominale inutile [5]. Son traitement médical, par antalgiques et anti-inflammatoires, évite une augmentation de la durée d’hospitalisation ainsi qu’une antibiothérapie inutile.

## Conflits d’intérêts

Les auteurs ne declarent aucun conflits d’intérêts.

## Contributions des auteurs

Dr Siham Alaoui Rachidi a interprété le scanner du patient, a réalisé une recherche bibliographique et a rédigé le manuscrit. Tous les auteurs ont lu et approuvé la version finale du manuscrit.
